# The role of general practice nurses in supporting people to quit smoking: A qualitative study

**DOI:** 10.1371/journal.pone.0306555

**Published:** 2024-07-18

**Authors:** Hannah Jongebloed, Eileen Cole, Emma Dean, Anna Ugalde

**Affiliations:** 1 Institute for Health Transformation, Faculty of Health, Deakin University, Burwood, Australia; 2 Quit Victoria, Cancer Council Victoria, East Melbourne, Australia; UV: Universiteti Ismail Qemali Vlore, ALBANIA

## Abstract

**Purpose:**

Encounters with General Practitioners (GPs) have previously been identified as opportune for the delivery of smoking cessation care however the role of nurses in general practice settings is unclear. This study aimed to understand how nurses are providing smoking cessation care in general practice.

**Methods:**

Participants were registered nurses currently working in a general practice setting in Australia, who participated in one-off interviews over Zoom. Interviews were recorded and a thematic analysis was conducted.

**Results:**

Fourteen nurses participated of which 13 (93%) were female. Three themes were evident in the data: 1) *Nurses’ current practices in supporting people to quit smoking*, *2) The influence of the general practice setting on smoking cessation discussions and 3) The challenges experienced by nurses in providing optimal smoking cessation care*. *Theme one* describes the strategies currently employed by nurses to deliver smoking cessation care such as identifying appropriate clinical scenarios to have smoking cessation conversations with patients. *Theme two* explores the impact of diversity in the systems, processes, and structures across Australian general practice settings on the support offered by nurses, such as opportunities for ongoing relationships with patients *Theme three* focuses on ambiguity in nurses’ roles within the practice setting including a lack of clarity for nurses in their roles in delivering smoking cessation care in the general practice setting.

**Conclusions:**

General practice nurses recognise the importance of their role in providing smoking cessation care and consider that general practice settings are ideally positioned to deliver that care. Smoking cessation care provided by nurses varies according to systems and processes within general practice clinics and relationships with general practitioners. Vaping is an emerging issue and nurses are seeking information on how to address this with patients. There is opportunity to support nurses to provide improved smoking cessation care.

## Introduction

Despite improvements in global smoking rates [1], smoking is still a prominent cause of death and disease in Australia [2]. Smoking cessation improves health outcomes for people with a range of chronic conditions including, cancer, cardiovascular disease and chronic respiratory disease [3, 4].

Best practice smoking cessation care consisting of advice, behavioural intervention and pharmacotherapy [5], should be routinely provided in health care settings. Advice delivered by health professionals can increase smoking cessation by patients [6] and models that guide delivery of smoking cessation care include the AAH (Ask, Advise, Help) model [5] or the 5As (Ask, Advise, Assess, Assist and Arrange) [7].

Asking about smoking status, advising all patients who smoke to quit and offering help through behavioural intervention (e.g. Quitline) and clinically appropriate pharmacotherapy should be offered to all people in general practice who smoke [8]. Both the AAH brief advice model and the comprehensive 5As approach for smoking cessation support can be used by clinicians to connect patients who smoke to this care [9]. These approaches can be effective in increasing the number of people who are able to achieve smoking cessation [10]. Recently updated provisional Australian guidance includes nicotine vaping products (NVPs), recommending that “For people who want to quit but have failed to achieve smoking cessation with first-line therapy (combination of behavioural support and TGA-approved pharmacotherapy), it may be reasonable to recommend NVPs in conjunction with behavioural support. The decision to proceed with this treatment must be part of an evidence-informed shared-decision making process” [11]. In Australia, a prescription is required to access NVPs legally. Currently there are no NVPs listed in the Australian Register of Therapeutic Goods (ARTG) [12].

Primary healthcare practitioners or general practitioners (referred to as general practitioners or GPs throughout) are often the first point of contact for patients with the healthcare system [13]. Encounters with GPs have previously been identified as an opportune time for the delivery of brief advice for smoking cessation [6]. Brief advice has been found to increase rates of cessation amongst patients [14] and smoking cessation interventions have been demonstrated to be more relatively more impactful in preventing death than other primary prevention activities delivered via primary care [15]. The Royal Australian College of General Practitioners guidelines on supporting smoking cessation for health professionals recommends all patients who smoke are offered brief advice in routine consultations or appointments, a system for identifying smokers and documenting tobacco use should be in place, and that follow-up is offered to all who are attempting to quit [16]. However, GPs often report barriers to delivering smoking cessation care to patients including time restraints as well as lack of skills, adequate education, and confidence [15, 17].

Primary health care nurses can successfully support people to stop smoking [10]. Nurses may supplement and increase the delivery of smoking cessation care to patients attending general practices. It is recognised that smoking cessation advice delivered by multiple health professionals can support a patient’s preparedness to quit [18], however upskilling practice nurses to provide smoking cessation care in addition to the GP is required [17]. Understanding how nurses can contribute to smoking cessation discussions is important in understanding optimal delivery of these interventions within general practice.

In Australia, the effectiveness smoking cessation care provided primarily by practice nurses has previously been explored [19]. Despite low uptake of the nurse delivered smoking cessation intervention in the randomised controlled trial, nurses interviewed were supportive of delivering smoking cessation advice [20]. While this suggests nurses agree that they have a role in assisting people with smoking cessation, there is a need to further understand how that role can be optimised. Internationally, work exploring nurses delivering smoking cessation care in primary care settings has also been conducted in the Netherlands [21, 22], South Korea [23], Spain [24, 25], the UK [26–34] and the USA [35–37].

The current study aimed to understand the nurses own perspectives of their current knowledge and practices regarding the provision of smoking cessation care, and their roles in providing that support within general practice settings. This knowledge is essential to optimising the nurses role.

## Materials and methods

### Study design

This study consisted of qualitative interviews with nurses across general practice settings in Australia.

Ethics approval for this project was obtained by Cancer Council Victoria (Clearance Number: HREC 2201).

### Participants and recruitment

A market research recruitment company was contracted to undertake participant recruitment for the project. Recruitment commenced on the 28^th of^ October 2022 and closed on the 30^th^ of November 2022. This included identification of eligible participants, scheduling of a suitable interview time and provision of the participant information and consent form, which was then returned to Deakin University researchers. Once the consent form was returned, participants were provided with a URL via email to join a Zoom meeting for the scheduled interview.

To be eligible to participate, prospective participants were required to be registered nurses currently working in a general practice setting in Australia. Recruitment aimed to include nurses from each state and territory, across metropolitan, regional and rural areas, with a range of ages and genders. An established framework was used to define and rural areas [38]. Prospective participants were considered ineligible if they had participated in market research on the topic of smoking cessation in the last 3 months or if they themselves or a member of their household had ever received funding and/or incentives from or worked for smoking cessation pharmaceutical companies, tobacco and/or e-cigarette/vaping industries.

Participants were offered compensation for their involvement.

### Data collection

An interview guide was developed by experienced qualitative researchers and reviewed by experts with content knowledge in the topic and peers with expertise in qualitative research; it was not formally piloted or evaluated. Interviews were scheduled with participants at a suitable time. Fourteen primary care nurses working in general practice settings participated in one-on-one interviews which were conducted by the first author (HJ) via Zoom.

The questions in the interview guide focused on nurses experiences providing smoking and vaping cessation care, and barriers and facilitators to providing this care, and minor changes were made to the questions as interviews progressed. Data collection and analysis were concurrent and recruitment ceased when no new themes were evident in the data.

### Analysis

The recording and transcription functions on Zoom were utilised. Transcripts were reviewed for accuracy and a thematic analysis was conducted whereby transcripts were reviewed and codes were applied to salient text. A coding structure was developed to establish key themes. Quotes are identified by participant number. Numbers were assigned randomly and are not an indication of the order interviews were conducted. One researcher (HJ) led the coding of the data analysis. A subset of interviews was reviewed by a second researcher (AU). In the event of discrepancy, codes were discussed until consensus was reached.

## Results

### Participants

Fourteen one-on-one interviews with primary care nurses working in general practice settings were conducted. Participants included 13 female and one male. Nurses were recruited across most states and territories with the exception of the two territories which were not represented (Northern Territory and Australian Capital Territory). Interviews ranged from 36 minutes to 56 minutes. Nurses varied in age and experience, ranging from 26–51 years in age and 1–28 years of experience in nursing. Three worked in a small GP practice (less than 5 GPs equivalent full time), five in a mid-sized practice (5–9 GPs equivalent full time) and six in a large practice (10 or more GPs equivalent full time). Two nurses identified their practice as being in a regional area, one in a rural area and the remaining 11 were in metropolitan areas.

### Interview data

Three themes were evident in the data. These included 1) nurses’ current practices in supporting people to quit smoking, 2) the influence of the general practice setting on smoking cessation discussions and 3) the challenges experienced by nurses in providing optimal smoking cessation care. Table 1 presents a summary of the three themes.

**Table 1:** Description of themes and key findings.

Nurses’ current practices in supporting people to quit smoking	The influence of the general practice setting on smoking cessation discussions	The challenges experienced by nurses in providing optimal smoking cessation care
• Nurses were supportive of initiating smoking cessation discussions • Clinical scenarios provided opportunities • Importance of providing individualised support	• Lack of time a commonly reported barrier • General practice setting provides unique opportunities • Ongoing patient relationships can be developed in general practice settings • Benefits of a collaborative approach	• Ambiguity in nurses’ roles within the practice setting • Vaping as an evolving and emerging issue and its use as a mechanism to quit smoking

#### Nurses’ current practices in supporting people to quit smoking

Nurses reflected upon the methods they use to provide cessation care in their current practice. This theme describes the provision of smoking cessation care by nurses in the context of using the Ask, Advise, Help (AAH) brief advice model.

Nurses described the clinical scenarios in which they considered it was appropriate to have a conversation with a patient about smoking cessation, and how they would approach that discussion. Several nurses noted the importance of approaching the conversation gently and that certain situations offered an opportunity for nurses to initiate a conversation about smoking cessation:

“So, if they had wound care, something like that. When you are a smoker, then we would definitely use that to help, educate them, understand their circulation.” (P1)

Some clinical circumstances were identified as not being appropriate to initiate a conversation about smoking cessation:

“Sometimes it can be overwhelming when a patient has so many health issues. I feel like I might not offer some health advice, because I don’t want to add to the burden of problems that this person has, or I feel like I’m not the right person to be bringing up a specific issue.” (P7)

Patient factors were also identified as negatively impacting:

“If the patient was aggressive or intimidating, I just wouldn’t. I mean, unless they were the one to bring it up.” (P4)

Most nurses interviewed shared the view that having multiple conversations with patients over time was an important part of delivering smoking cessation care, but some felt repetition was not beneficial. Notably nurses identified the importance of following up with patients who had expressed an interest in quitting:

“I think it’s an ongoing conversation because that certainly can be a first step, but it’s certainly not the last—that can’t be the last. No, you should say ‘catch up with me in a fortnight and see how you’re going’, or ‘if you want to see me before then, you can’, and we always make a follow up GP appointment too. I always make sure I go walk them to the front desk and say, we’re booking another appointment, and they’re like ‘Oh, no, I don’t need to see the doctor, I will be right, I’ll give it a go’, No, no, I really want to see you in a fortnight, and it gives us some accountability, because they know they’ve got to come back.” (P5)

Most nurses shared the perspective that asking about smoking status was standard at a practice level however, the delivery of smoking cessation care varied depending on practitioner experience and training. Nurses described the practice of providing individualised support by assessing the patients preferred approach to quitting and discussing with the patient a range of possible smoking cessation treatment options. Use of nicotine replacement therapy (NRT) and referral to Quitline were identified as key components of discussions. Several nurses described a tendency to refer to NRT or medication, without an explicit discussion about behavioural interventions:

“As primary care nurses we know of the gums and the lozenges, and the patches, and hypnotherapy, or even medication. There’s talking to your doctor about that. We know about the Quitline.” (P3)

“But I’d say, like, get pushing the patches and getting them started on the treatment would be where I’m like focusing my attention, if someone really wanted to quit.” (P1)

#### The influence of the general practice setting on smoking cessation discussions

The unique general practice structure was recognised as influential for an individual nurse’s current practice, as well as for the delivery of smoking cessation care across the practice. Whilst nurses felt that the general practice setting offered an opportunity to deliver smoking cessation care, they acknowledged that some practice systems may act as barriers to nurses routinely providing smoking cessation care such as time pressure:

“So, unless there allotted a proper time, there’s not much opportunistic time and sort of, I think, to get into smoking. You really do need like that half an hour appointment to really, yeah, be able to touch on that.” (P2)

It was generally agreed that, compared to nursing in other settings, general practice nursing provided a unique opportunity to deliver smoking cessation care. Additionally, one nurse noted that there is a trust that exists between patients and nurses in the general practice setting:

“Smoking cessation is the biggest here [in this setting]. It’s because here, in general practice, I think we’ve got more ongoing care. In the hospital and home setting you saw someone for such a short period of time.” (P1)

“I think there is more privacy in a GP clinic setting, more opportunity, you know, especially in those health assessments.” (P8)

Nurses also felt that a collaborative approach, where multiple health care practitioners were contributing to numerous conversations with patients about smoking cessation was important:

“I think it’s the, it’s collaborative because nurses have a different way of talking to than doctors, and vice versa. It only, sometimes just comes down to the way someone says something that makes all the difference. It might be the exact same messages there. But someone just says something differently that hits them the right way that they’re like, yeah, this is something I need to do.” (P14)

Use of medical record systems to record a patient’s smoking status and document smoking cessation discussions was considered to be an important part of the nurse’s role:

“We try to make sure we’re up in the 90’s [90%]. All our patients have that record on their file and we share that record on their file.” (P1)

#### The challenges experienced by nurses in providing optimal smoking cessation care

While nurses often acknowledged their role in smoking cessation care (Theme 1) and reflected on how the system may support this (Theme 2), several key challenges were recognised about optimising their role as a provider of smoking cessation care. Two key challenges are presented in this theme: the differences between nursing and GP roles and vaping as a new and emerging issue.

The nurse’s role was seen as different to that of GP’s in supporting patients to quit smoking. Many participants felt uncertain about how they could support smoking cessation in general practice clinics while this was also the GPs role. Some nurses felt limited in what advice and support they could provide, recognising that GPs often are more knowledgeable, or these discussions align better with their roles:

“Their best bet would be to see a GP I think…especially if they’ve been smoking a long time. Smoking’s an addiction and they need to be supported with that and if they’re supported, they’ve got a better chance of quitting and not relapsing.” (P10)

Participants noted that different GPs had different expectations, and this influenced the role they had in providing cessation support:

“It depends on the doctor itself. we have a couple of young doctors who are all over it. A couple of our old doctors talk to the nurse–‘Here’s a script, talk to the nurse’. And so, then we do—for those particular doctors who are just, or some of is just because they’re time-poor usually. But sometimes it’s just… handballing the job.” (P5)

Many participants reflected on the differences between the interactions GPs and nurses had with patients. Several acknowledged that the nature of the interactions with patients for nurses meant that ongoing relationships could be more difficult to build, and smoking discussions were less appropriate.

In recognising their role in smoking cessation care, some nurses felt their role was to work with the GP, rather than the patient. This allowed them to leverage the GP’s experience. Many felt their role was to support the GP:

“We don’t go too much into the plan, the GP does. But it would be like a step-down plan, and obviously prescriptions from the GP and they will follow that. And then we would assist in following that up… That’s usually the conversation the GP would have and then we’re there just to support that conversation.” (P4)

Many people noted that in the context of their work, their role in supporting smoking cessation wasn’t always clear. Ambiguity around the nurse’s role in supporting smoking cessation in patients led many participants to reflect that they had inadequate training. Several participants recognised that there were gaps in the knowledge which they felt impacted their ability to deliver smoking cessation care to patients:

“I think if my knowledge was broader, then I could compliment more what the Quitline are also trying to help people with.” (P1)

Some participants recognised vaping as an evolving and emerging issue for which they felt they lacked knowledge and skills to address. Many participants reflected that they did not address vaping in the same way they addressed smoking and most lacked the confidence to discuss vaping:

“Whereas I think with smoking like you sort of have a bit more knowledge in linking it to heart disease or linking it to like liver or lung function. But you can’t just say vaping is bad for you, you should quit.” (P2)

“Vaping is a real knowledge lapse on our practices or on me, personally. I wouldn’t have a confident conversation based on vaping, other than what I’ve seen physically. I just don’t know enough about it." (P7)

Most nurses did not accept vaping as an appropriate mechanism to quit smoking and noted this wasn’t recommended to patients. Participants also observed that it was unusual for health professionals to recommend vaping as a means to quit smoking. Given that endorsing vaping was unusual, it was also evident that nurses held gaps in knowledge regarding the processes in general practice settings to support vaping as an option to quit smoking:

“Certainly not with the support of a GP, not our GPs…I know patients that have been vaping instead. I don’t know if they have the support of their GP, I don’t think that would probably be encouraged.” (P3)

“I haven’t come across it [prescription for vaping], I have heard of it, but I don’t really know a lot about it.” (P6)

## Discussion

This study explored the role of the practice nurses in providing cessation care. Three key themes were evident in the qualitative data: nurses’ current practices in supporting people to quit smoking, influence of the setting and the challenges of optimising nurse led smoking cessation care. Findings indicate nurses want to support people to quit smoking and adopt a range of strategies to facilitate this.

While this study showed support from nurses to engage in smoking cessation discussions, the diversity of the practices was also evident. This is likely to vary across individual practices but also with setting, for example, differences are evident between rural and metropolitan general practice clinics, with rural areas facing unique challenges around smaller and dispersed populations and more distance to access healthcare [39]. Nurses in this study reported even within practices, individual practitioners have their own preferences and approaches to discussing smoking cessation. Individual practitioner factors and perspectives represent one domain of the multitude of determinants known to influence the delivery of evidence-based care, such as patient, organisations and system-level factors [40]. Understanding these factors, models of care and opportunities for providing smoking cessation care may be an important future step in this program of work. We found nurses want support in delivering smoking cessation care, understanding how this can be optimised is an important next step.

While this was one of the first studies to understand how general practice nurses discuss smoking cessation, literature has explored GP views. An interview study found GPs reported that brief advice is efficient and easy to implement [41]. A cross sectional survey found GPs were one of the better implementers of brief advice compared to other clinical groups [42]. A recent conversation analytic study consisted of analysis of real patient-GP interactions [19]. This analysis of 31 consultations found that doctors initiated the topic of smoking in many cases (25/31 consultations), though linking of smoking to the patient’s medical condition could be met with resistance [43]. Analysis of nurse-patient conversations about smoking cessation would add to the findings of our study and is a potential avenue for future work.

Vaping was an emerging and prominent issue that was an area of concern and ambiguity. A systematic review on GP’s views of vaping as a smoking cessation tool found mixed results, with some GPs recommending it as a cessation tool and others did not feel it was a suitable cessation tool [44]. Consistent with our study however, nurses noted that from their observations in general practice settings, GPs lacked knowledge and confidence in having discussions around e-cigarettes. Our interviews showed that nurses held concerns around vaping as an emerging problem more broadly and they wanted further knowledge on addressing this with patients directly. There is an absence of guidance on how general practices can advise patients on this emerging issue and these studies suggest guidance is needed to inform both GPs and nurses on how to address vaping.

There are some limitations to be considered in interpreting these results. The general practice setting was a relevant factor, yet we collected very limited information about types of these settings, patient throughput or models of care. Given the diversity of general practice settings, understanding these may be an important avenue for both qualitative and quantitative work. During recruitment we did not screen potential participants for tobacco use. Research has shown that nurses who smoke themselves are less likely to administer smoking cessation care [45] and this would be an important consideration for future work. Additionally, nurses work across a range of healthcare settings and a comparison of their role in the delivery of smoking cessation care across different settings could be explored in further work. We recruited a small sample size however were able to have representation across metropolitan, regional and rural settings. This is one of the few studies that has focused on understanding the current practices and potential for the role of nurses who work in general practice settings to support people to quit. Understanding this involvement can drive improved smoking cessation interventions to lead to quitting and associated health benefits. Future work could understand how to optimise smoking cessation discussions across different models of care, adopting conversational analysis to understand interactions between nurses and patients about smoking cessation and the development and dissemination of guidance to inform general practice staff on how to address vaping.

General practice nurses recognise cessation care as an important part of their role and general practice settings are ideally positioned to deliver that care. Smoking cessation care varies with different clinics and practitioners. Vaping is an emerging issue and nurses are seeking information on how to address this with patients. Smoking cessation delivery by nurses in general practice settings holds potential and there is opportunity to support nurses to provide improved smoking cessation care.
